# Dynamic Analysis of the Abnormal Isometric Strength Movement Pattern between Shoulder and Elbow Joint in Patients with Hemiplegia

**DOI:** 10.1155/2018/1817485

**Published:** 2018-02-11

**Authors:** Yali Liu, Yuezhen Hong, Linhong Ji

**Affiliations:** ^1^Division of Intelligent and Biomechanical System, State Key Laboratory of Tribology, Tsinghua University, Haidian, Beijing, China; ^2^National Key Laboratory of Human Factors Engineering, China Astronaut Research and Training Center, Haidian, Beijing, China

## Abstract

Patients with hemiplegia usually have weak muscle selectivity and usually perform strength at a secondary joint (secondary strength) during performing a strength at one joint (primary strength). The abnormal strength pattern between shoulder and elbow joint has been analyzed by the maximum value while the performing process with strength changing from 0 to maximum then to 0 was a dynamic process. The objective of this study was to develop a method to dynamically analyze the strength changing process. Ten patients were asked to perform four group asks (maximum and 50% maximum voluntary strength in shoulder abduction, shoulder adduction, elbow flexion, and elbow extension). Strength and activities from seven muscles were measured. The changes of secondary strength had significant correlation with those of primary strength in all tasks (*R* > 0.76, *p* < 0.01). The antagonistic muscles were moderately influenced by the primary strength (*R* > 0.4, *p* < 0.01). Deltoid muscles, biceps brachii, triceps brachii, and brachioradialis had significant influences on the abnormal strength pattern (all *p* < 0.01). The dynamic method was proved to be efficient to analyze the different influences of muscles on the abnormal strength pattern. The muscles, deltoid muscles, biceps brachii, triceps brachii, and brachioradialis, much influenced the stereotyped movement pattern between shoulder and elbow joint.

## 1. Introduction

Hemiplegia, including the characteristics such as weakness and decreased selective motor control, is a common clinical sequelae following a stroke [1]. Hemiplegia is associated with a reduced number of functional corticospinal and corticobulbar fibers to the spinal and brainstem because of lesions in the cerebral cortex.

Deficits in the control of independent joint movements have been reported, especially the abnormal torque pattern and the abnormal muscle coordination patterns [2–5]. Researchers analyzed the abnormal joint torque pattern to explore the characteristics of the symptom and what caused the symptom [3, 6–8]. Dewald and Beer studied the abnormal joint torque patterns using a six-freedom load cell [9]. They pointed out that the strong abnormal implicated torque pattern existed in the conjunct movement of shoulder abduction during elbow flexion and shoulder adduction during elbow extension. Bohannon and Smith tested the muscle strength by a manual hand-held dynamometer and found that there were muscle strength imbalances in hemiplegic patients [10]. These studies focused on the mean or the peak of the torque and strength at shoulder and elbow joint by considering the torque and strength as a static value, and the muscle activation and torque pattern were separately analyzed [11]. The static analysis method for the strength and few analyses of relationship between strength and muscle activation limit our knowledge on the characteristics of the abnormal patterns. The dynamic analysis between the kinematic or dynamic parameters and the muscle activation has been reported to be beneficial to understand patients' movement relationship between the extrinsic characteristics and the inherent nature [12]. Therefore, a dynamic analysis on the performing process is needed to explore the relationship between the strength and muscle activation and investigate the elements influencing the abnormal strength patterns.

The main purpose of this study is to explore the strength patterns between shoulder and elbow joint and develop the relationship between strength patterns and muscle activation. Our hypothesis is that the strength performing is a dynamic process with the strength changing from 0 to maximum and then to 0. The strength patterns could be generated by some specific muscles during primary strength actions and the primary strength could have different influences on different muscles at the secondary joint.

## 2. Methods

### 2.1. Subjects

Ten stroke patients with unilateral hemiplegia in the upper extremity were recruited. Most patients (7/10) had lesions at the basal ganglia on the right hemisphere and were evaluated by Fugl-Meyer Assessment scores for upper limb (Table 1). The criteria for recruitment in this experiment were (1) the first onset of stroke, diagnosed with definite lesions on hemisphere by CT or MRI; (2) able to understand experimenter's request; (3) age between 30 and 80 years; (4) no lesions on cerebellum and brainstem; (5) no severe inflammation, pathological injury, and malformation in the paretic arm; (6) no severe visual impairment; and (7) no acute conditions.

All subjects were provided the informed consent form for the experiment, which was approved by the medical ethics committee.

### 2.2. Isometric Strength Measurement Instrument Description

**Figure 1:**
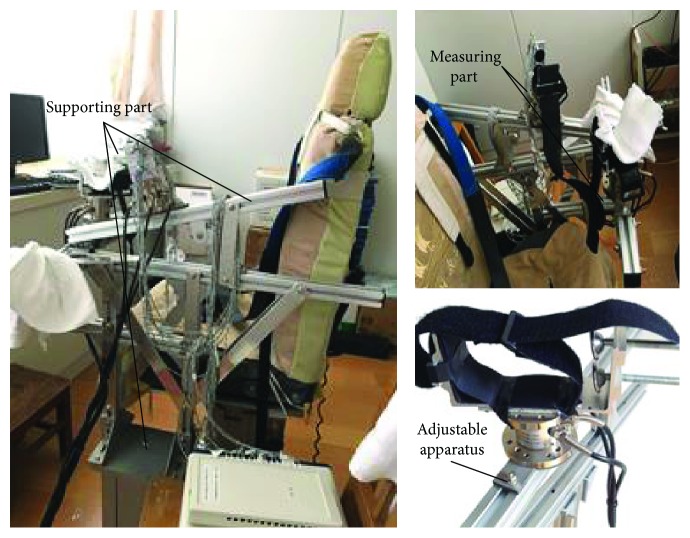
The main parts of ISMI.

Isometric strength measurement instrument (ISMI) was used to measure the shoulder and elbow strength. The mechanical apparatus comprises of two main parts (i.e., supporting part and the measuring part) including two three-freedom force sensors (Figure 1). The supporting part, which is made of aluminum alloy, consists of a U frame, two inclined beams, and a weight support part. The U frame is used to fix the position of the force sensors and the inclined beams are used to enhance the structure when users perform tasks. The weight support part is used to keep the apparatus' balance when users perform actions. The measuring part is used to measure the strength at the segment's center of mass. The positions of centers of mass (COM) of segments are decided according to the human anatomy (Table 2) [13].

### 2.3. Experiment Protocol

**Figure 2:**
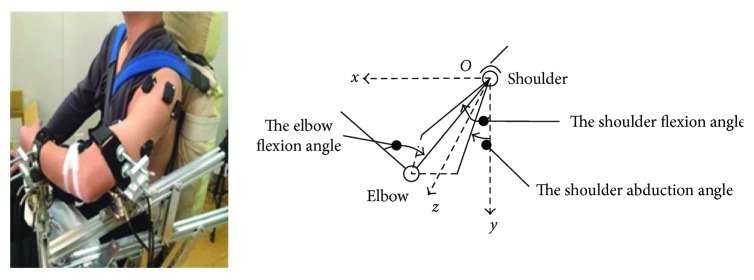
The subject's posture during experiment. The *x,O,y* coordinate plane represented the sagittal plane, the *y,O,z* coordinate plane represented the coronal plane, and the *x,O,z* coordinate plane represented the transection plane. The shoulder flexion angle was defined by the angle between the projection of upper limb on the sagittal plane (the *x,O,y* coordinate plane) and the coordinate axes (the *y*-axes). The shoulder abduction angle was defined by the angle between the projection of upper limb on the coronal plane (the *y,O,z* coordinate plane) and the coordinate axes (the *y*-axes). The elbow flexion angle was defined by the angle between the forearm and upper limb on the plane determined by the forearm and upper limb.

Each subject performed four group strength tasks including maximum and 50% maximum voluntary strength in shoulder abduction (MVS-ABD and 50% MVS-ABD), shoulder adduction (MVS-ADD and 50% MVS-ADD), elbow flexion (MVS-FLEX and 50% MVS-FLEX), and elbow extension (MVS-EXT and 50% MVS-EXT). Each subject was asked to sit in the chair (Figure 1) with his/her trunk fixed to restrict trunk movement and maintain his/her shoulder 75° abduction and 40° flexion as well as elbow neutral flexion at 90° (Figure 2). The strength was defined by the force detected by the force sensor at the segment's center of mass in Table 2.

**Figure 3:**
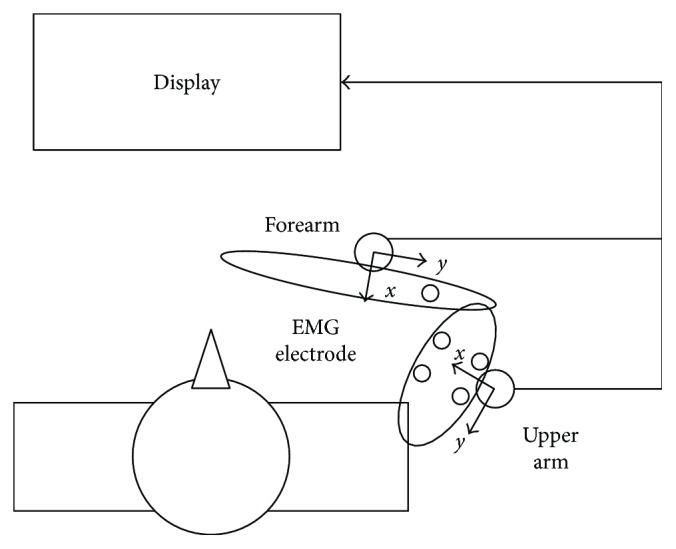
The schematic design for forces and EMG measured. The force in the X positive direction at the forearm meant the force in elbow flexion direction and the force in the X positive direction at the upper arm meant the force in shoulder adduction direction.

The strength at shoulder and elbow joint and electromyographic (EMG) signals from pectoralis major (PM), anterior, intermediate, and posterior deltoid (AD, MD, and PD), biceps brachii (BB), triceps brachii (TB), and brachioraialis (BR) were recorded during each task (Figure 3). The strength was measured at 10 Hz by two force sensors (Baisen, China). Real-time visual feedback of the strength value was shown to the subject in the display instrument (XSR90 color paperless recorder, Baisen, China). EMG signals were measured by active differential electrodes (Delsys, 16-channel Bagnolis EMG System, Boston, MA, USA) attached on muscle bellies with 1 cm interelectrode distance after cleaning the skin. EMG signals were sampled at 2000 Hz.

First, each subject performed maximum voluntary strength in each group. Then the maximum strength value was used to calculate the target strength in fifty percent voluntary strength task. Each strength task lasted 1-2 s and the subject can adjust the performing strength according to the displayer mentioned earlier. Each task was repeated three times. EMG signals and strength signals were measured simultaneously by a synchronous trigger using a continuous high-level signal from the same control computer.

### 2.4. Data Processing

#### 2.4.1. Dynamic Analysis of Strength Patterns

The primary strength was defined as the strength which subjects intended to make maximum and the secondary strength was defined as the strength at the adjacent joint during performing the primary strength.

The strengths of each task were calculated using MATLAB (Matlabworks R2014a). In each trail, subjects performed the primary strength from 0 to the target and then to 0. The overall effort of the performing task lasted 1-2 s, which was a dynamic process. The changing strength was the extrinsic characteristic of different muscle contraction. The dynamic process can make it clear to understand the relationship between the strength and the muscle contraction in patients with hemiplegia.

For each task, strength magnitude was calculated by averaging values with a moving 250 ms window. The maximum voluntary strength (MVS) was determined by the maximum strength value in MVS task. All strength values in the 50% MVS task were normalized by the corresponding MVS according to equation (1). The process of strength performing task in the 50% MVS task was normalized to 101 points by the entire duration of the task in order to compare the subjects' performance.

(1) $$\begin{array}{c} F_{normalized}=\frac{F_{50\%task}}{{MVS}_{task}} \end{array}$$

*F*_normalized_ meant the normalized strength value in the 50% MVS task; *F*_50%task_ meant the strength values in the 50% MVS tasks; and MVS_task_ meant the maximum strength value in the corresponding MVS task.

#### 2.4.2. Analysis of EMG Signals

The raw EMG signals were removed baseline drift and rectified. Then, a fourth-order Butterworth band-pass filter with cutoff values between 10 Hz and 500 Hz was applied [14–16]. The root mean square was calculated with a moving 100 ms window to smooth the signals [12, 17]. All rectification, filtering, and smoothing were processed in the software (EMGworks 4.1.7, Analysis). Afterwards, MATLAB (Matlabworks R2014a) was used to extract the EMG signals of each muscle in the corresponding strength performing duration. The EMG signals in the 50% MVS task were normalized by the maximum value in the corresponding MVS task according to equation (2). The EMG signals in the 50% MVS task were normalized to 101 points by the entire duration of the task.

(2) $$\begin{array}{c} R_{muscle\left(i\right)}=\frac{A_{50\%muscle\left(i\right)}}{Amax\left(A_{maxmuscle\left(i\right)}\right)} \end{array}$$

Muscle(*i*) (*i* = 1,2,3…7) meant the seven different muscles: pectoralis major, anterior, intermediate, and posterior deltoid, biceps brachii, triceps brachii, and brachioraialis. *A*_50%muscle(*i*)_ meant the EMG amplitudes of muscle(*i*) in the 50% MVS task and Amax(*A*_maxmuscle(*i*)_) meant the EMG maximum amplitude of muscle(*i*) in the MVS task.

#### 2.4.3. Influence of Muscles on Strength Patterns

The process of subjects' performing strength tasks was considered as a dynamic process. The changes of primary strength and secondary strength were the extrinsic characteristics of muscle contraction. A regression analysis was conducted to analyze the relationship between secondary strength in primary strength tasks and muscle activation.

### 2.5. Statistical Analysis

Statistical analysis of the strengths and EMG signals was processed in the software IBM SPSS Statistics 22. General linear regression and ridge regression were used to analyze the different influences of muscle activation on secondary strength patterns. The coefficients of muscles described the degree of the muscle influences on the strength patterns.

We used correlation analysis to explore the correlation between the primary strength and secondary strength as well as activation of muscles at secondary joints. Bivariate Pearson correlation with two-tailed test was carried out to analyze (i) the dynamic relationship between the primary strength and the secondary strength and (ii) the relationship between primary strength and muscles at secondary joints. Statistical significance in all of the statistical analysis was set at *p* < 0.05.

## 3. Results

### 3.1. Dynamic Analysis of Strength Patterns

**Figure 4:**
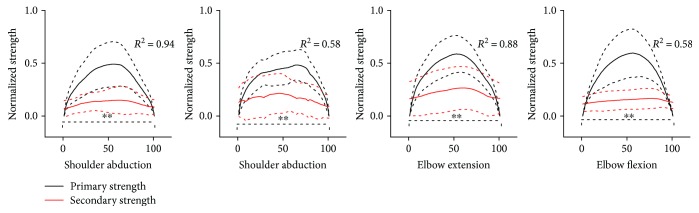
The variation of secondary and primary strength during primary strength actions. The horizontal axis in Figure 4 represented the total effort of the primary strength and the vertical axis represented the normalized strength of secondary and primary strength in 50% MVC tasks. The black curves represented the normalized primary strength and the red ones represented the normalized secondary strength. The solid curves represented the average values among different subjects and the dashed curves represented one standard deviation among different subjects. ^∗∗^*p* < 0.01.

Primary strength at shoulder or elbow joint changed from 0 to 50% MVS and then to 0 during 50% MVS tasks. The variation of secondary strength was in high correlation to the variation of primary strength with all correlation coefficients above 50%, especially elbow flexion during shoulder abduction and shoulder adduction during elbow extension with the correlation coefficients above 85% (Figure 4).

In 50% MVS-ABD, 50% MVS-ADD, 50% MVS-FLEX, and 50% MVS-EXT task, all variations of secondary strength were in high correlations to the variations of primary strength (*R*^2^ > 50%). It should be highlighted that the correlation coefficients of elbow flexion during shoulder abduction and shoulder adduction during elbow extension were above 85% (Figure 4).

### 3.2. Relationship between the Strength Patterns and Muscle Activation

Table 3 illustrated the results of the linear regression analysis for the relationship between the strength patterns and muscle activation during the dynamic strength changing process. There existed a significant linear relationship (*R*^2^ > 0.70) between primary strength and the activation of muscles at secondary joint in each task, which indicated that muscles at secondary joint can be activated in primary strength task, especially TB, BR activation in shoulder abduction, BB, TB, BR activation in shoulder adduction, AD, MD, PD activation in elbow extension, and PM, AD, MD, PD activation in elbow flexion.

The correlation coefficients by the multilinear regression analysis reflected the influence degree of the independent variable on the dependent variable. Muscle activation influenced the strength, and the degree of the influence can be described by the correlation coefficients. There was a significant linear relationship between secondary strength and muscles at primary joint via ridge regression analysis in each task (50% MVS-ABD *R*^2^ = 0.70, 50% MVS-ADD *R*^2^ = 0.72, 50% MVS-FLEX *R*^2^ = 0.94 and 50% MVS-EXT *R*^2^ = 0.65, Table 4).

## 4. Discussion

Most of the previous quantitative studies of upper limb strength and muscle activation were analyzed separately [1, 9, 10], and the strength pattern was just analyzed by an averaged value. However, the strength pattern was a dynamic process. The process of the strength changes during actions cannot be described by a certain average value and the muscles which have more influence on the secondary strength pattern cannot also be determined by a single value. In this study, participants were asked to perform the dynamic process through the strength changing from 0 to target values and then to 0 again. A multiple linear regression method was used to analyze the relationship between the strength and muscle activation and explore which muscle activation primarily influencing the abnormal pattern.

### 4.1. The Dynamic Process of Strength Patterns

For subjects with hemiplegia, secondary strengths were in significant correlations with primary strengths in the conjunct movement of shoulder abduction during elbow flexion and elbow extension during shoulder adduction, which was compatible with the abnormal joint torque patterns found in 2000 by Dewald and Beer [9]. Furthermore, it can be found that the secondary strength was in a higher correlation to the primary strength in shoulder abduction and elbow extension with both of the correlation coefficients above 0.95. The phenomenon may be demonstrated by the common stereotyped flexor synergies in upper limb, which was consistent with the Roh et al. study [5]. The higher correlation coefficients reflected that patients can perform a stronger secondary strength during performing primary strength in shoulder abduction and elbow extension. The remarkably increasing elbow flexion strength along with shoulder abduction could be explained by patients generating a large strength at the distal joint to compensate for the weakness of shoulder [18, 19]. The increasing elbow flexion may decrease the mechanical inertial torque of upper limb and make it easier for subjects with weak shoulder strength to perform shoulder abduction.

The increasing shoulder adduction along with elbow extension demonstrated the rationale of the motor learning method in Bobath concept [20, 21]. Patients could generate a large strength at the proximal joint in order to guarantee the stabilization of the shoulder during performance at distal joint. Besides, patients generated an opposite torque at the upper arm by increasing shoulder adduction during elbow extension, thus decreasing the total torque.

### 4.2. Relationship between Muscle Activation and Secondary Strength

Patients with hemiplegia usually have lesions in their cerebral cortex. They generally have reduced corticospinal input to shoulder and distal arm muscles. The reduction from corticospinal control signals results in an increased dependence on residual brainstem descending pathways (such as vestibulospinal, reticulospinal, and rectospinal pathways) [22], which may activate extensive branching and innervate more neurons over spinal segments. Accordingly, the increased dependence on brainstem pathways may induce coactivation of more muscles, thus altering the strength pattern in patients with hemiplegia [23]. Muscles at secondary joint were influenced by the activation of muscles at primary joint because of the decreased control of muscle selectivity [24].

However, all of the muscles at secondary joint were influenced at different degrees. The antagonistic muscles at the elbow joint were much influenced during the primary strength at shoulder joint. For example, TB, the antagonistic muscle for elbow flexion, was much influenced during shoulder abduction; BB and BR, the antagonistic muscles for elbow extension were much influenced during shoulder adduction. Besides, the antagonistic muscles at shoulder joint were also much influenced by primary strength at elbow joint. Primary strength in elbow extension had much influence on PD and MD (the antagonistic muscles for shoulder adduction) and primary strength in elbow flexion had much influence on AD (the antagonistic muscles for shoulder abduction). These results may also reflect the declined inhibition on the antagonistic muscles for patients with hemiplegia [25–27].

The dynamic analysis of muscle activation and secondary strength demonstrates the different degrees of different muscles' influence on the secondary strength. The significant level of each muscle's influence on the normalized secondary strength illustrates how the secondary strength patterns depend on the muscle activation. Secondary strength pattern of shoulder abduction during elbow flexion is much influenced by shoulder abduction agonist and antagonist (MD and PD). Secondary strength pattern of shoulder adduction during elbow extension is significantly influenced by shoulder adduction muscles (PM, AD, and PD). Elbow extension muscles (TB and BR) have significant effects on secondary strength pattern of elbow extension during shoulder adduction. Elbow flexion muscles (BB, TB, and BR) have significant effects on secondary strength pattern of elbow flexion during shoulder abduction.

An important limitation of this study is that it only explored the secondary strength patterns between shoulder abduction/adduction and elbow flexion/extension, while shoulder flexion/extension and shoulder internal/rotation may also influence elbow flexion/extension in subjects with hemiplegia. The designed isometric strength measurement instrument for the upper limb may have influence on the activation of muscles at proximal and distal segments. Finally, the sample size of investigated subjects was small and the patients were not in the same rehabilitation state: some were in subacute stroke and others were in chronic stroke. In the future, we should recruit more numbers of patients in the same rehabilitation state and design more test postures in the experiment.

## 5. Conclusions

The process of strength performing is a dynamic process with strength changing from 0 to target values and then to 0 again, even in an isometric strength task. This study conducted a dynamic analysis of the abnormal secondary strength pattern in patients with hemiplegia by using multiple linear analysis. It can be concluded that secondary strength in elbow flexion during shoulder abduction and shoulder adduction during elbow extension was in high correlation with the primary strength. It also suggests that patients may intend to decrease the total torque by generating an opposite torque at shoulder or elbow joint during elbow or shoulder strength tasks. Deltoid, biceps brachii, triceps brachii, and brachioradialis have more influences on the abnormal movement pattern.

## Figures and Tables

**Table 1:** The clinical data of hemiplegic stroke patients.

Item	Patients with hemiplegia (*N* = 10)
Age (years)	53.80 ± 13.32
Gender, male/female	8/2
Lesion location, left/right hemisphere	1/9
Days since stroke	76.0 ± 43.9
FMA score for UL	21.1 ± 9.4

FMA score for UL, Fugl-Meyer Assessment scores for upper limb (maximum score = 66).

**Table 2:** The ratio of COM position of segments.

Segments	Gender	Lcs	Lcx
Upper limb	M	47.8	52.2
	F	46.7	53.3
Forearm	M	42.4	57.6
	F	45.3	54.7

COM: center of mass; M: male; F: female; Lcs: the ratio of the length from COM position to the proximal point on the total length of the segment; Lcx: the ratio of the length from COM position to the distal point on the total length of the segment.

**Table 3:** Pearson correlation coefficients between the activation of muscles at secondary joint and primary strength.

Primary strength	Pearson correlation coefficients of variables							
	Secondary strength	*R*_PM_	*R*_AD_	*R*_MD_	*R*_PD_	*R*_BB_	*R*_TB_	*R*_BR_
ABD	FLEX	—	—	—	—	−0.07	−0.42^∗∗^	−0.59^∗∗^
ADD	EXT	—	—	—	—	0.61^∗∗^	0.30^∗∗^	0.83^∗∗^
EXT	ADD	−0.08	0.94^∗∗^	0.95^∗∗^	0.45^∗∗^	—	—	—
FLEX	ABD	0.67^∗∗^	0.77^∗∗^	0.54^∗∗^	0.75^∗∗^	—	—	—

ABD: strength at shoulder abduction; ADD: strength at shoulder adduction; EXT: strength at elbow extension; FLEX: strength at elbow flexion. *R*_muscles_ (muscles = PM, AD, MD, PD), the standardized coefficients between the strength at elbow and the activation of muscles (pectoralis major(PM), anterior deltoid (AD), intermediate deltoid (MD), posterior deltoid (PD)); *R*_muscles_ (muscles = BB, TB, BR), the standardized coefficients between the strength at shoulder and the activation of muscles (biceps brachii (BB), triceps brachii (TB), brachioraialis (BR)). ^∗∗^*p* < 0.01.

**Table 4:** Multiple regression analysis of normalized secondary strength and muscles at primary joint.

Normalized second strength	Primary strength direction	Standardized coefficients of independent variables							*R* ^2^
		*R*_PM_	*R*_AD_	*R*_MD_	*R*_PD_	*R*_BB_	*R*_TB_	*R*_BR_	
FLEX	ABD	0.13^∗∗^	0.14^∗∗^	0.29^∗∗^	0.32^∗∗^	—	—	—	0.70^∗∗^
EXT	ADD	0.33^∗∗^	0.33^∗∗^	0.04	0.28^∗∗^	—	—	—	0.72^∗∗^
ADD	EXT	—	—	—	—	−0.07^∗^	1.12^∗∗^	0.19^∗∗^	0.94^∗∗^
ABD	FLEX	—	—	—	—	−0.30^∗∗^	0.49^∗∗^	0.45^∗∗^	0.65^∗∗^

ABD: strength at shoulder abduction; ADD: strength at shoulder adduction; EXT: strength at elbow extension; FLEX: strength at elbow flexion. *R*_muscles_ (muscles = PM, AD, MD, PD), the standardized coefficients between the strength at elbow and the activation of muscles (pectoralis major(PM), anterior deltoid (AD), intermediate deltoid (MD), posterior deltoid (PD)); *R*_muscles_ (muscles = BB, TB, BR), the standardized coefficients between the strength at shoulder and the activation of muscles (biceps brachii (BB), triceps brachii (TB), brachioraialis (BR)). ^∗^0.01 < *p* < 0.05; ^∗∗^*p* < 0.01.

## References

[1] Neckel N., Pelliccio M., Nichols D, Hidler J (2006). Quantification of functional weakness and abnormal synergy patterns in the lower limb of individuals with chronic stroke. *Journal of Neuroengineering and Rehabilitation.*.

[2] Bosecker C., Dipietro L., Volpe B., Krebs H. I. (2010). Kinematic robot-based evaluation scales and clinical counterparts to measure upper limb motor performance in patients with chronic stroke. *Neurorehabilitation and Neural Repair*.

[3] Metrot J., Mottet D., Relave I. (2011). Bimanual coordination in stroke recovery: kinematic analysis provides open leads to individualize upper limb rehabilitation. *Annals of Physical and Rehabilitation Medicine*.

[4] Wagner J. M., Lang C. E., Sahrmann S. A., Edwards D. F., Dromerick A. W. (2007). Sensorimotor impairments and reaching performance in subjects with poststroke hemiparesis during the first few months of recovery. *Physical Therapy*.

[5] Roh J., Rymer W. Z., Perreault E. J., Yoo S. B., Beer R. F. (2013). Alterations in upper limb muscle synergy structure in chronic stroke survivors. *Journal of Neurophysiology*.

[6] Ettema G. J. C., Taylor E., North J. D., Kippers V. (2005). Muscle synergies at the elbow in static and oscillating isometric torque tasks with dual degrees of freedom. *Motor Control*.

[7] Ada L., O'Dwyer N., O'Neill E. (2006). Relation between spasticity, weakness and contracture of the elbow flexors and upper limb activity after stroke: an observational study. *Disability and Rehabilitation*.

[8] Lum P. S., Burgar C. G., Shor P. C. (2004). Evidence for improved muscle activation patterns after retraining of reaching movements with the MIME robotic system in subjects with post-stroke hemiparesis. *IEEE Transactions on Neural Systems and Rehabilitation Engineering*.

[9] Dewald J. P. A., Beer R. F. (2001). Abnormal joint torque patterns in the paretic upper limb of subjects with hemiparesis. *Muscle & Nerve*.

[10] Bohannon R. W., Smith M. B. (1987). Assessment of strength deficits in eight paretic upper extremity muscle groups of stroke patients with hemiplegia. *Physical Therapy*.

[11] Hong Y.-z., Sui J.-f., Ji L.-h., Xi L., Sheng B. (2015). Force characteristics of synergistic movement between shoulder and elbow joints. *Chinese Journal of Rehabilitation Theory and Practice*.

[12] Guan X., Liu Y., Gao L. (2016). Trunk muscle activity patterns in a person with spinal cord injury walking with different un-powered exoskeletons: a case study. *Journal of Rehabilitation Medicine*.

[13] China Standards Press (1998). *Inertial Parameters of Adult Human Body*.

[14] Chen S.-K., Wu M.-T., Huang C.-H., Wu J.-H., Guo L.-Y., Wu W.-L. (2013). The analysis of upper limb movement and EMG activation during the snatch under various loading conditions. *Journal of Mechanics in Medicine and Biology*.

[15] Rogowski I., Rouffet D., Lambalot F., Brosseau O., Hautier C. (2011). Trunk and upper limb muscle activation during flat and topspin forehand drives in young tennis players. *Journal of Applied Biomechanics*.

[16] Nagai K., Yamada M., Uemura K., Yamada Y., Ichihashi N., Tsuboyama T. (2011). Differences in muscle coactivation during postural control between healthy older and young adults. *Archives of Gerontology and Geriatrics*.

[17] Konrad P. (2005). *The ABC of EMG: a practical introduction to kinesiological electromyography*.

[18] Mercierand C., Bourbonnais D. (2004). Rlative shoulder fexor and handgrip strength is related to upper limb function after stroke. *Clinical Rehabilitation*.

[19] Smith M. (2015). Neurological rehabilitation: optimizing motor performance. *Physiotherapy Canada*.

[20] Raine S., Meadows L., Lynch-Ellerington M. (2013). *Bobath Concept: Theory and Clinical Practice in Neurological Rehabilitation*.

[21] Kollen B. J., Lennon S., Lyons B. (2009). The effectiveness of the Bobath concept in stroke rehabilitation: what is the evidence?. *Stroke*.

[22] Lemon R. N. (2008). Descending pathways in motor control. *Annual Review of Neuroscience*.

[23] Kuypers H. G. J. M. (1964). The descending pathways to the spinal cord, their anatomy and function. *Progress in Brain Research*.

[24] Staudt M., Grodd W., Gerloff C., Erb M., Stitz J., Krägeloh-Mann I. (2002). Two types of ipsilateral reorganization in congenital hemiparesis: a TMS and fMRI study. *Brain*.

[25] Gowland C., deBruin H., Basmajian J. V., Plews N., Burcea I. (1992). Agonist and antagonist activity during voluntary upper-limb movement in patients with stroke. *Physical Therapy*.

[26] Levin M. F., Selles R. W., Verheul M. H. G., Meijer O. G. (2000). Deficits in the coordination of agonist and antagonist muscles in stroke patients: implications for normal motor control. *Brain Research*.

[27] Silva A., Sousa A. S. P., Tavares J. M. R. S., Tinoco A., Santos R., Sousa F. (2012). Ankle dynamic in stroke patients: agonist vs. antagonist muscle relations. *Somatosensory & Motor Research*.

